# Ombitasvir/paritaprevir/ritonavir plus ribavirin for 24 weeks in patients with HCV GT4 and compensated cirrhosis (AGATE‐I Part II)

**DOI:** 10.1002/hsr2.92

**Published:** 2019-03-01

**Authors:** Tarik Asselah, Negar Niki Alami, Christophe Moreno, Stanislas Pol, Stylianos Karatapanis, Michael Gschwantler, Yves Horsmans, Ioannis Elefsiniotis, Dominique Larrey, Carlo Ferrari, Mario Rizzetto, Alessandra Orlandini, Jose Luis Calleja, Savino Bruno, Gretja Schnell, Roula Qaqish, Rebecca Redman, Tami Pilot‐Matias, Sarah Kopecky‐Bromberg, Yao Yu, Niloufar Mobashery

**Affiliations:** ^1^ Hopital Beaujon Clichy France; ^2^ AbbVie, Inc Chicago Illinois; ^3^ CUB Hôpital Erasme Université Libre de Bruxelles Brussels Belgium; ^4^ Université Paris Descartes Paris France; ^5^ Hepatology Department Cochin Hospital, APHP Paris France; ^6^ INSERM U1223, UMS‐20 and Center for Translational Science Institut Pasteur Paris France; ^7^ Department of Internal Medicine General Hospital of Rhodes Rhodes Greece; ^8^ Medizinische Abteilung mit Gastroenterologie, Hepatologie, Endoskopie und Ambulanz Wilhelminenspital Wien Austria; ^9^ Cliniques Universitaires Saint‐Luc, Department of Gastroenterology Université catholique de Louvain Brussels Belgium; ^10^ Academic Department of Internal Medicine‐Hepatology Unit, General Oncology Hospital of Kifisia “Agioi Anargyroi” National and Kapodistrian University of Athens Athens Greece; ^11^ Liver Unit‐IRB‐INSERM1040 Hôpital Saint Eloi Montpellier France; ^12^ Università degli Studi di Parma Parma Italy; ^13^ University of Torino Turin Italy; ^14^ Azienda Ospedaliero–Universitaria di Parma Parma Italy; ^15^ Liver Unit, Puerta de Hierro University Hospital, Instituto de Investigación Sanitaria Puerta de Hierro‐Majadajonda, Centro de Investigación Biomédica en Red de Enfermedades Hepáticas y Digestivas Majadahonda Madrid Spain; ^16^ Department of Biomedical Sciences Humanitas University, Humanitas Research Hospital Milan Italy; ^17^ Pharmacyclics LLC Sunnyvale California USA

**Keywords:** compensated cirrhosis, DAAs, genotype 4, HCV

## Abstract

**Background and Aims:**

AGATE‐I Part I previously reported high sustained virologic response rates in hepatitis C genotype 4 patients with cirrhosis, with 12 and 16 weeks' treatment with a combination of two direct‐acting antivirals, ombitasvir and paritaprevir (codosed with ritonavir), plus ribavirin. Part II, reported here, extended the trial to include a 24‐week treatment arm to fully assess treatment duration in patients with chronic hepatitis C genotype 4 infection and compensated cirrhosis.

**Methods:**

Enrollment took place between June and November of 2015. Treatment‐naive and interferon‐experienced patients with chronic hepatitis C genotype 4 infection and compensated cirrhosis were enrolled into Arm C; patients previously treated with a sofosbuvir‐based regimen were enrolled into Arm D. All patients received a 24‐week treatment with ombitasvir, paritaprevir, and ritonavir plus ribavirin. The primary outcome was the proportion of patients with a sustained virologic response (hepatitis C virus RNA < 25 IU/mL) at posttreatment week 12 in the intention‐to‐treat population. The safety population included all patients who received at least one dose of study drug.

**Results:**

In total, 64 patients were enrolled into AGATE‐I Part II. Sustained virologic response at posttreatment week 12 was achieved in 57 of 61 patients (93.4%; 97.5% confidence interval, 92.6‐97.7) in Arm C and 3 of 3 patients (100%) in Arm D. Two patients were missing SVR12 data, and two prematurely discontinued treatment. The most common adverse events for Arm C were fatigue (16 [26%]) and asthenia (15 [25%]). Results were comparable with those reported in Part I.

**Conclusions:**

AGATE‐I Part II indicates that extending treatment beyond 12 weeks in genotype 4–infected patients with compensated cirrhosis does not offer additional benefit.

## Abstract 2

Perspective on this article: Direct‐acting antiviral therapy for chronic HCV genotype 4 infection: exploring new regimens

**List of abbreviations**
AASLD: American Association for the Study of Liver Disease
ALT: alanine aminotransferase
ANCOVA: analysis of covariance
AST: aspartate aminotransferase
CI: confidence interval
DAA: direct‐acting antiviral
EASL: European Association for the Study of the Liver
HCV: hepatitis C virus
SVR12: sustained virologic response 12 weeks after the end of treatment
ULN: upper limit of normal

## 1 INTRODUCTION

Infection with hepatitis C virus (HCV) is associated with substantial morbidity and mortality, and affects more than 185 million people worldwide.^1^, ^2^ HCV genotype 4 accounts for more than 8% of total HCV cases (more than 90% of HCV infections in Egypt alone),^1^ and its prevalence is characterized by wide regional variations, with the highest prevalence reported for the Middle East and sub‐Saharan Africa.^1^ An increase in distribution of HCV genotype 4 infections has been noted globally, because of effects of migration, with several European studies reporting 9% to 14% of HCV infections^1^, ^3^ attributable to genotype 4. HCV genotype 4 demonstrates substantial genetic variability, with 17 confirmed subtypes.^4^, ^5^ In Egypt, genotype 4a is the predominant subtype reported,^6^, ^7^ whereas Saudi Arabia and parts of Europe have high rates of subtypes^4^, ^5^ 4a, 4c, and 4d.

Treatment guidelines from the European Association for the Study of the Liver (EASL) and the American Association for the Study of Liver Disease (AASLD) now include recommendations for several all‐oral direct‐acting antiviral (DAA) regimens for patients with HCV genotype 4 infection,^8^, ^9^ including once‐daily ombitasvir (an NS5A inhibitor), coformulated with paritaprevir (an NS3/4A protease inhibitor identified by AbbVie and Enanta) and the pharmaco‐enhancer ritonavir, plus twice‐daily ribavirin. The safety and efficacy of the all‐oral direct‐acting antiviral (DAA) regimen ombitasvir/paritaprevir/ritonavir plus ribavirin was first established in the phase 2b PEARL‐I study, in which this combination given for 12 weeks achieved sustained virologic response 12 weeks after the end of treatment (SVR12) in all 91 treatment‐naive (n = 42) and treatment‐experienced (n = 49) patients with HCV genotype 4 infection without cirrhosis.^10^, ^11^

Although there are recommended regimens for patients with HCV genotype 4 infection with compensated cirrhosis, these patients are often underrepresented in clinical trials.^12^, ^13^, ^14^, ^15^, ^16^, ^17^ More extensive efficacy and safety data are needed in this patient population, particularly to address the unresolved issue of appropriate treatment duration in these patients. The phase 3 AGATE‐I trial was the first large, prospective clinical trial to study the efficacy and safety of an investigational agent in HCV genotype 4 patients with compensated cirrhosis across a set of three treatment durations.^18^ Part I of the trial, previously published,^18^ randomized 120 patients to 12 or 16 weeks of treatment with ombitasvir/paritaprevir/ritonavir plus ribavirin, and demonstrated SVR12 rates of 97% and 100% (the latter previously reported as 98% because one patient was initially lost to follow‐up but has since returned to clinic and achieved SVR), respectively.^18^ Here, we report the results of Part II, which expanded the scope of the AGATE‐I trial to include a 24‐week treatment group in order to identify the optimal treatment duration for HCV genotype 4 patients with compensated cirrhosis. Additionally, a study arm for patients with treatment history of virologic failure with sofosbuvir/pegylated interferon plus ribavirin or sofosbuvir plus ribavirin was included to explore efficacy and safety of ombitasvir/paritaprevir/ritonavir plus ribavirin in these patients with limited treatment options.

## 2 PATIENTS AND METHODS

### 2.1 Study design and patients

AGATE‐I is a multinational, partly randomized, phase 3, open‐label trial (http://clinicaltrials.gov, NCT02265237). Patients with HCV genotype 4 infection and compensated cirrhosis were recruited from academic, public, and private hospitals and clinics in Austria, Belgium, Canada, France, Germany, Italy, Spain, and the United States. The study was designed to have four arms, with Part I (Arms A and B) randomizing patients to 12 or 16 weeks of treatment.^18^ Enrollment into Part II (Arms C and D) began after randomization in Part I was completed (full eligibility criteria can be found in the appendix p2).

Patients in Arm C were either treatment naive or previously treated with interferon plus ribavirin or pegylated interferon plus ribavirin. Prior treatment responses included null responders, partial responders, and relapsers (full definitions of prior treatment experience are given in the appendix p5). A minimum of 10 null responders were to be enrolled to ensure adequate representation of historically harder‐to‐treat patients.

Patients to be enrolled into Arm D were previously treated with sofosbuvir and pegylated interferon plus ribavirin or sofosbuvir plus ribavirin and were classified as either prior breakthrough/nonresponders or prior relapsers (full definitions are given in the appendix p5). All prior therapy must have been completed no fewer than 2 months prior to the screening visit. Complete inclusion and exclusion criteria are available in the appendix (p2).

The study was designed accordingly to Good Clinical Practice guidelines, the 1975 Declaration of Helsinki, and applicable regulations, with institutional review board approval for all study sites. A list of the International Ethics Committees can be found in the supporting information. All patients provided written informed consent prior to enrollment.

### 2.2 Procedures

All patients received two tablets of coformulated oral ombitasvir (25 mg), paritaprevir (150 mg), and ritonavir (100 mg), taken with food once daily, plus a total daily oral dose of 1000 mg ribavirin if the patient's bodyweight was less than 75 kg, or 1200 mg if bodyweight was 75 kg or more, taken in two doses daily for 24 weeks. All patients who received at least one dose of study drugs were monitored for 48 weeks after last dose of treatment. Full details on ribavirin dose modification protocols, RNA quantification, genotyping, and phylogenetic analysis are available in the appendix (pp6‐7).

### 2.3 Outcomes

The primary efficacy end point for Part II was the proportion of patients who achieved SVR12 (HCV RNA less than lower limit of quantification at posttreatment week 12) in Arm C.

The secondary outcomes for Part II were to assess the proportion of patients with either on‐treatment virologic failure or posttreatment relapse and to compare the proportion of patients achieving SVR12 between Arms B and C. The number and percentage of patients with treatment‐emergent adverse events (any event that begins or worsens in severity after initiation of study drug through 30 days poststudy drug dosing) were tabulated by severity and by relationship to study drug. Laboratory variables were assessed throughout the study. Further information on definitions of relapse and virologic failure and additional outcomes and monitoring is available in the appendix (p8).

### 2.4 Statistical analysis

The study was designed to test the hypothesis that percentages of treatment‐naive and interferon‐experienced HCV genotype 4–infected patients with compensated cirrhosis treated with ombitasvir/paritaprevir/ritonavir coadministered with ribavirin for 12, 16, and 24 weeks achieving SVR12 were superior to the historical rate of 67% (appendix p9). Full details of all statistical analyses carried out are available in the appendix (p9).

## 3 RESULTS

In Part II of the AGATE‐I study, 64 patients were enrolled between June 10, 2015, and November 19, 2015: 61 patients to Arm C and 3 patients to Arm D. All patients were assigned to receive 24 weeks of treatment. All patients received at least one dose of study drug.

Arm C included 30 (49%) treatment‐experienced patients, of whom 13 (21%) were null responders, 7 (11%) were partial responders, and 10 (16%) were relapsers to prior treatment. All three patients in Arm D had virologic relapse after prior treatment with sofosbuvir plus ribavirin with or without pegylated interferon. Sequence analysis identified 33 (52%) of 64 patients in Part II infected with subtype 4a (one with a mixed infection with subtype 1a), 14 (22%) with 4d, and 17 (27%) with other genotype 4 subtypes. Full patient demographics and baseline disease characteristics are summarized in Table 1.

**Table 1:** Patient demographics and baseline characteristics

	Arm C (n = 61)	Arm D (n = 3)
Sex, n (%)		
Male	43 (71%)	3 (100%)
Female	18 (30%)	…
Age (y), median (range)	55 (18‐78)	67 (53‐67)
Ethnicity,^a^ n (%)		
White	45 (74%)	2 (67%)
Black or African American	14 (23%)	…
Asian	2 (3%)	…
Native Hawaiian or Pacific Islander	…	1 (33%)
Hispanic or Latino	6 (10%)	…
BMI (kg/m^2^), mean ± SD	27.8 ± 4.9	32.8 ± 9.2
HCV genotype 4 subtype, n (%)		
4^b^	3 (5%)	0
4a	32 (52%)	0
4a/1a	1 (2%)	0
4b	2 (3%)	0
4c	1 (2%)	0
4d	12 (20%)	2 (67%)
4f	1 (2%)	0
4h	1 (2%)	0
4k	4 (7%)	0
4l	1 (2%)	0
4n	1 (2%)	0
4o	1 (2%)	1 (33%)
4p	0	0
4q	0	0
4r	1 (2%)	0
4t	0	0
Missing	0	0
*IL28B* non‐CC genotype, n (%)	53 (88%)	1 (33%)
HCV RNA (log_10_ IU/mL), mean ± SD	6.1 ± 0.5	5.2 ± 1
HCV ≥ 800,000 IU/mL, n (%)	41 (68%)	…
Interferon or ribavirin treatment experience, n (%)		
Treatment naive	31 (51%)	0
Null responder	13 (21%)	…
Partial responder	7 (12%)	…
Relapser	10 (16%)	…
Prior sofosbuvir therapy relapser	n/a	3 (100%)
Child Pugh score^c^		
5	57 (95%)	3 (100%)
6	3 (5%)	0
FibroTest score,^d^ mean ± SD	0.7 ± 0.2	0.9 ± 0.03
Hemoglobin concentration (g/dL), median (range)	14.9 (11.4‐18.5)	15.5 (14‐16.3)
Albumin concentration (g/L), median (range)	42 (29‐51)	38 (36‐40)
Platelet count (×10^9^/L), median (range)	157 (58‐340)	104 (100‐172)
α‐Fetoprotein (ng/mL), median (range)	8.1 (1‐81)	10 (8.3‐12)

Abbreviations: BMI, body mass index; HCV, hepatitis C virus; *IL28B*, interleukin‐28B gene; SD, standard deviation.

Data are n (%), median (range), or mean (SD).

a Ethnicity is self‐reported.

b Unable to subtype.

c Child‐Pugh scores are those reported at baseline. None of these patients had any clinical decompensation episodes throughout the study.

d Based on observations from 59 patients from the Arm C and 3 patients from Arm D.

**Figure 1:**
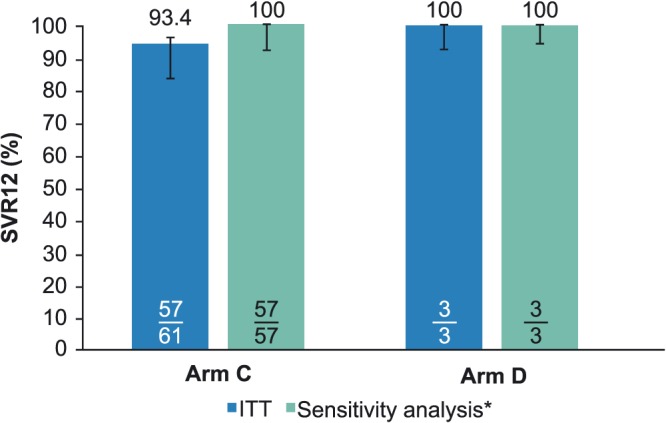
Efficacy of ombitasvir, paritaprevir, and ritonavir plus ribavirin in patients with hepatitis C virus genotype 4 infection and compensated cirrhosis. Data are percentage for the SVR12 ITT analysis; bars represent 97.5% confidence intervals for each arm as calculated by the Wilson score method. ITT, intention‐to‐treat population; SVR12, sustained virologic response at posttreatment week 12. ^*^Sensitivity analysis excluded patients who were categorized as either having “prematurely discontinued study drug with no on‐treatment virologic failure” or “missing follow‐up data in the SVR12 window”

SVR12 was achieved in 57 of 61 patients (93.4%; 97.5% confidence interval [CI], 82.6‐97.7) in Arm C and all three patients in Arm D (100%; Figure 1). In Arm C, patients not achieving SVR12 were two patients with missing SVR12 data and two patients with premature discontinuation of treatment: one patient due to an adverse event of acute liver toxicity and one due to unknown reasons. Superiority to historical rates achieved with pegylated interferon plus ribavirin was shown in Arm C because the lower bound of the 97.5% CI for SVR12 was higher than the predefined threshold (67%).

**Figure 2:**
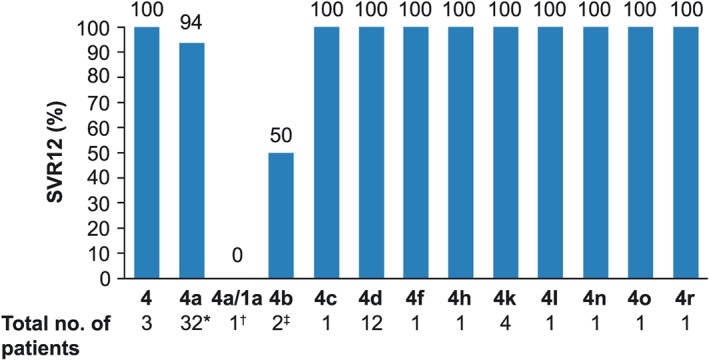
Efficacy of ombitasvir, paritaprevir, and ritonavir plus ribavirin in patients with hepatitis C virus genotype 4 infection and compensated cirrhosis by subtype. ^*^One patient was lost to follow‐up and one prematurely discontinued study drug; ^†^patient prematurely discontinued study drug; ^‡^one patient was lost to follow‐up

Comparison of SVR12 by HCV genotype 4 subtypes revealed that the percentages of patients achieving SVR12 for all subtypes were consistent with those of the overall intention‐to‐treat population, with no clinically meaningful differences between subgroups (Figure 2).

A sensitivity analysis excluding patients who did not achieve SVR12 for reasons other than virologic failure (eg, early discontinuation or missing SVR12 data) showed SVR12 was achieved in 57 of 57 patients (100%; 95% CI, 93.7‐100) in Arm C and in all three patients in Arm D (100%; Figure 1).

Comparison of SVR12 rates from the 24‐week treatment Arm C with Arm B using the stratum‐adjusted Mantel‐Haenszel method showed that no significant difference was observed in SVR12 between patients receiving treatment for either 12 (Arm A) versus 16 weeks (Arm B), or 16 versus 24 weeks (Arm C; Figure S1, appendix p11).

Small improvements in FibroTest score were observed posttreatment in all treatment arms in Part II. Arm C mean changed from 0.71 at baseline to 0.59 at posttreatment week 12; and Arm D mean changed from 0.91 at baseline to 0.83 at posttreatment week 12. In AGATE‐I Part I, Arm B mean changed from 0.69 at baseline to 0.60 at posttreatment week 12. No significant difference in mean change between Arms B and C (*P* = 0.37, analysis of covariance [ANCOVA]) was identified.

Overall, 54 (89%) of 61 patients in Arm C experienced at least one treatment‐emergent adverse event (Table 2), the most common being fatigue (n = 16 [26%]) and asthenia (n = 15 [25%]). Three patients (5%) had severe adverse events, with none attributed by the investigator as being related to study drugs. Three (5%) patients in total experienced serious adverse events (full details in the appendix p12). One patient in Arm C was admitted to hospital in treatment week 3 of the study with grade 4 acute liver toxicity, which had reasonable possibility of being related to study drugs. The patient had prolonged elevations of total bilirubin (direct and indirect) ranging from 65 to 96 μmol/L (grade 3; ≥3 × upper limit of normal [ULN]) from day 9 of treatment with concomitant increase in alanine aminotransferase (ALT) elevations of 166 to 219 U/L (grade 3; >5 × ULN) from day 15, resulting in discontinuation of study drug on day 17. ALT and total bilirubin levels normalized posttreatment. This patient did not achieve SVR.

**Table 2:** Adverse events and postbaseline laboratory abnormalities (safety population)

	Arm C (n = 61)	Arm D (n = 3)
Adverse events		
Any adverse events	54 (89%)	3 (100%)
Adverse events leading to study drug discontinuation	2 (3%)	0
Severe adverse event	3 (5%)	0
Serious adverse event	3 (5%)	0
Death	0	0
Adverse events occurring in >10% of patients^a^		
Fatigue	16 (26%)	0
Asthenia	15 (25%)	1 (33%)
Headache	13 (21%)	0
Anemia	7 (11%)	0
Pruritus	12 (20%)	1 (33%)
Nausea	5 (8%)	1 (33%)
Hemoglobin decreased	4 (7%)	0
Dizziness	0	0
Insomnia	5 (8%)	1 (33%)
Myalgia	3 (5%)	0
Postbaseline laboratory abnormalities, n/N_OBS		
Hemoglobin ≥ grade 2 (<10 g/dL)	7/61 (11%)	0/3
Hemoglobin ≥ grade 3 (<8 g/dL)	0/61	0/3
Alanine aminotransferase ≥ grade 3 (>5 × ULN)	3/61 (5%)	0/3
Aspartate aminotransferase ≥ grade 3 (>5 × ULN)	1/61 (2%)	0/3
Total bilirubin ≥ grade 3 (≥3 × ULN)	5/61 (8%)	0/3

Abbreviation: ULN, upper limit of normal.

a Adverse events occurring in >10% of patients in Arm C; n/N_OBS, indicates the number of patients with postbaseline value through the final treatment value for the respective parameter.

Postbaseline laboratory abnormalities (regardless of baseline level) for patients in Arm C are presented in Table 2. Grade 2 hemoglobin reductions (<10 g/dL) occurred in 7 (11%) of 61 patients, and no patient experienced a grade 3 or 4 reduction in hemoglobin (<8 g/dL; Table 2). In total, three patients experienced a grade 3 (>5 × ULN) elevation in ALT levels (Table 2). One patient had an isolated incident of ALT elevations at posttreatment week 1 (223 U/L). Two patients had prolonged elevations: The first patient (also described above) experienced acute liver toxicity with ALT elevations 166 to 219 U/L from day 15 to day 17, and the second patient experienced ALT elevations 248 to 383 U/L from weeks 2 to 6, which resulted in the patient discontinuing the study drug. ALT elevations were resolved by end of treatment in the second patient and were not associated with concomitant increases in total bilirubin. One patient (1.6%) had a grade 3 (>5 × ULN) aspartate aminotransferase (AST) elevation. Postbaseline changes in total bilirubin >3 × ULN (grade 3) were reported for five (8%) patients and were mostly indirect. No grade 4 laboratory abnormalities were reported.

The safety profile from Part II was comparable with Part I with similar rates of adverse events and serious adverse events^18^ (full results reported in the appendix p13).

All three patients in Arm D experienced at least one treatment‐emergent adverse event. All events were mild in severity. No patient in Arm D experienced a serious adverse event or discontinued study drugs because of an adverse event. No grade 2 or greater hemoglobin (<10 g/dL) abnormalities were detected. None of the three patients in Arm D reported any grade 3 and above laboratory abnormalities.

Fifteen (23%) of 64 patients (all in Arm C) in Part II had modifications to their ribavirin dosage. The most frequent cause of dose modification was a decrease in hemoglobin, reported in 8 (13%) of the 64 patients. Two patients (3%) reduced ribavirin dosage because of decreases in creatinine clearance, and eight (13%) patients reduced because of other reasons. One patient who had a decrease in creatinine clearance switched to alternate daily doses of ribavirin because of renal impairment. All patients were managed solely with ribavirin dose modifications, with none requiring erythropoietin (use permitted at the discretion of the investigator) or transfusions. Fourteen (93%) of 15 patients who had ribavirin dose reduction achieved SVR12. The one patient who did not achieve SVR12 was the patient that prematurely discontinued the study in treatment week 3 because of acute liver toxicity.

## 4 DISCUSSION

The efficacy and safety of all‐oral interferon‐free combination regimens for treatment of HCV genotype 4 infection, particularly in cirrhotic patients, have not been well characterized. The AGATE‐I study is the first large clinical study to evaluate interferon‐free treatment for patients with compensated cirrhosis infected with HCV genotype 4, with a total of 184 patients recruited globally across Parts I and II, ensuring a large patient diversity and representation of several genotype 4 subtypes. In Part II, presented here, patients received ombitasvir/paritaprevir/ritonavir plus ribavirin for 24 weeks, with 93% of patients achieving an SVR12. These results were comparable with the results from Part I, in which 97% of patients in the 12‐week group and 100% of patients in the 16‐week group achieved SVR12,^18^ with no significant differences observed in SVR12 between patients receiving treatment for 12 versus 16 weeks or 16 versus 24 weeks (Figure S1, appendix p11). Part II also included a study arm for patients with treatment history of prior virologic failure with sofosbuvir with pegylated interferon plus ribavirin or sofosbuvir plus ribavirin to assess the regimen as a possible retreatment option for such patients that have limited approved retreatment options for HCV genotype 4 infection. Although only three patients were enrolled in this arm, all achieved SVR12. No on‐treatment virologic failures or treatment relapses were reported in Part II, and only one patient was reported to have experienced virologic breakthrough^18^ in Part I, demonstrating a strong indication of efficacy of the regimen. Numerically, more patients experienced nonvirologic treatment failure in Part II (n = 4) than in Part I (n = 1), which may be a factor of longer treatment duration.

HCV genotype 4 has previously been considered difficult to treat because of variable efficacy between GT4 subtypes, with multiple studies reporting higher SVR rates in HCV GT4a–infected patients than in HCV GT4d–infected patients.^5^, ^19^, ^20^ Comparison of SVR12 by HCV genotype 4 subtypes in Arm C revealed that the percentage of patients achieving SVR12 for all subtypes represented in the study were consistent with those of the overall population, including 100% SVR12 in HCV GT4d–infected patients. Similar results were reported^18^ in Part I.

Comparison of FibroTest score across the three treatment durations showed slightly improved scores after treatment, and treatment duration longer than 12 weeks with ombitasvir/paritaprevir/ritonavir plus ribavirin did not lead to greater improvements, including the 24‐week arm presented in this study. HCV treatment can potentially reduce fibrosis and lead to regression of cirrhosis in selected patients.^21^, ^22^ Future studies with longer follow‐up periods will be vital to assess DAA treatment with improved clinical outcomes and to rule out any short‐term reduction in FibroTest score because of transient effects of HCV treatment on serum biomarkers (eg, gamma‐glutamyl transferase).^23^

The treatment‐emergent adverse events observed in Part II were generally mild and manageable, with only two patients discontinuing treatment in the 24‐week treatment arm because of a treatment‐related adverse event. One patient experienced prolonged elevations of ALT levels, and the other experienced grade 4 acute liver toxicity. In the latter case, concomitant increases in ALT and total bilirubin levels to grade 3 were reported; however, levels returned to normal after study drug was discontinued. Transient bilirubin levels were in part attributed to increases in indirect bilirubin, which is consistent with the inhibition or organic anion‐transporting protein 1B1 and 1B3 by paritaprevir and can also be attributed to ribavirin use.^24^ Twenty‐three percent of patients in Part II modified ribavirin dosage, with the most common cause for this being hemoglobin decrease, with no effect on overall SVR rates.

AGATE‐II, a companion study to AGATE‐I, examined the effect of ombitasvir/paritaprevir/ritonavir plus ribavirin in patients with HCV genotype 4 infection with cirrhosis in Egypt.^25^ Of those treated for 12 weeks, 97% (30 of 31) achieved SVR12, and in the 24‐week group, 93% (27 of 29) reached SVR12, consistent with the results reported in AGATE‐I. However, few data from other trials are available on the efficacy and safety of treatment in patients with HCV genotype 4 and compensated cirrhosis, with on average less than a quarter of patients with compensated cirrhosis enrolled in these previous trials.^26^, ^27^, ^28^, ^29^ Despite these relatively small numbers of cirrhotic patients in the majority of studies, high SVR rates were achieved in this population. At the time this study was conducted, EASL and AASLD guidelines recommended treatment of genotype 4 with compensated cirrhosis: ombitasvir/paritaprevir/ritonavir plus ribavirin, ledipasvir/sofosbuvir with or without ribavirin, sofosbuvir/velpatasvir, and elbasvir/grazoprevir with or without ribavirin.^8^, ^9^

Part II of the AGATE‐I trial demonstrated high SVR rates of the regimen with treatment for 24 weeks in HCV genotype 4–infected patients with compensated cirrhosis. Safety analyses from Parts I and II suggest that all treatment durations were similar and generally well tolerated, with only two patients in the 24‐week treatment groups discontinuing study drugs because of an adverse event. A limitation of the study is that it was not blinded or placebo controlled. However, using a placebo‐controlled design would be inappropriate in patients with compensated cirrhosis because of the potential risk of progression to decompensated liver disease if active treatment is delayed.

Part II of the AGATE trial indicates that extending treatment with ombitasvir/paritaprevir/ritonavir and ribavirin to 24 weeks in genotype 4–infected patients with compensated cirrhosis does not offer additional benefit to treatment efficacy or short‐term regression of fibrosis.

## FUNDING

AbbVie funded the study (nct02265237); contributed to study design; and participated in the collection, analysis, and interpretation of data, and preparation and approval of this report. All authors had access to all relevant study data, reviewed and approved the final report, and take full responsibility for the accuracy of the data and statistical analysis. The corresponding author contributed to the study design, had full access to all relevant study data, and had final responsibility for the decision to submit for publication.

## CONFLICTS OF INTEREST

N.N.A., G.S., R.R., T.P.‐M., S.K.‐B., Y.Y., and N.M. are employees of AbbVie and may hold stock or stock options.

T.A.: Clinical Investigator/Speaker/Consultant: AbbVie, Boehringer Ingelheim, BMS, Gilead Sciences, Janssen Pharmaceuticals, Merck Sharp & Dohme, Roche

C.M.: Research grants: AbbVie, Gilead Sciences, Janssen; Consultant: AbbVie, Gilead Sciences, Janssen, Merck Sharp & Dohme, BMS

S.P.: Consultant/Lecturer: BMS, Boehringer Ingelheim, Janssen, Gilead, MSD, Novartis, AbbVie; Grant/Research support: BMS, Gilead, Roche, MSD

S.K.: Research grant: AbbVie

M.G.: Advisor/Speaker: Janssen, MSD, BMS, Gilead, AbbVie; Grants: Gilead, AbbVie, MSD

Y.H.: AbbVie, Gilead, Janssen, MSD, BMS

I.E.: Advisor/Speaker: Gilead, AbbVie, MSD, BMS; Research grants: Gilead, AbbVie

D.L.: Participation in clinical studies: AbbVie

C.F.: Consultant: AbbVie, Gilead, Arrowhead, Humabs, MSD, Chiesi, Abivax; Research grants: Janssen, Cilag, Roche, BMS, Gilead, AbbVie

M.R.: Participation in clinical studies: AbbVie

A.O.: Consultant: AbbVie, MSD

J.L.C.: Consultant and Lecturer: AbbVie, BMS, MSD, Gilead Sciences

S.B.: Participation in clinical studies: AbbVie

R.Q.: Participation in clinical studies: AbbVie

## AUTHOR CONTRIBUTIONS

Conceptualization: Niloufar Mobashery

Data Curation: Yao Yu

Formal Analysis: Yao Yu

Investigation: Tarik Asselah, Negar Niki Alami, Christophe Moreno, Stanislas Pol, Stylianos Karatapanis, Michael Gschwantler, Yves Horsmans, Ioannis Elefsiniotis, Dominique Larrey, Carlo Ferrari, Mario Rizzetto, Alessandra Orlandini, Jose Luis Calleja, Savino Bruno, Gretja Schnell, Roula Qaqish, Tami Pilot‐Matias, Sarah Kopecky‐Bromberg

Methodology: Yao Yu

Supervision: Rebecca Redman, Niloufar Mobashery

Visualization: Negar Niki Alami

Writing – Original Draft: Negar Niki Alami

Writing – Review & Editing: Tarik Asselah, Negar Niki Alami, Christophe Moreno, Stanislas Pol, Stylianos Karatapanis, Michael Gschwantler, Yves Horsmans, Ioannis Elefsiniotis, Dominique Larrey, Carlo Ferrari, Mario Rizzetto, Alessandra Orlandini, Jose Luis Calleja, Savino Bruno, Gretja Schnell, Roula Qaqish, Rebecca Redman, Tami Pilot‐Matias, Sarah Kopecky‐Bromberg, Yao Yu, Niloufar Mobashery

All authors had access to all relevant study data, reviewed and approved the final report, and take full responsibility for the accuracy of the data and statistical analysis. The corresponding author contributed to the study design, had full access to all relevant study data, and had final responsibility for the decision to submit for publication.

## WRITING ASSISTANCE

Medical writing support was provided by Claire Wormleighton, PhD, of Medical Expressions, funded by AbbVie.

## Supporting information

Figure S1. Comparison of efficacy of ombitasvir, paritaprevir, and ritonavir, plus ribavirin, in patients with hepatitis C virus genotype 4 infection and compensated cirrhosis in Parts I and II.
Table: Adverse events and post‐baseline laboratory abnormalities (safety population)
