# Supplementary material for: Ombitasvir/paritaprevir/ritonavir plus ribavirin for 24 weeks in patients with HCV GT4 and compensated cirrhosis (AGATE‐I Part II)
Source: Health Sci Rep. 2019 Mar 1;2(3):e92. doi: 10.1002/hsr2.92 (PMC6427060; doi:10.1002/hsr2.92)
Supplement: Supplementary file 1 — Figure S1. Comparison of efficacy of ombitasvir, paritaprevir, and ritonavir, plus ribavirin, in patients with hepatitis C virus genotype 4 infection and compensated cirrhosis in Parts I and II. Table: Adverse events and post‐baseline laboratory abnormalities (safety population) [file HSR2-2-e92-s001.docx]

**SUPPLEMENTARY APPENDIX**

**Ombitasvir, paritaprevir, and ritonavir plus ribavirin for 24 weeks in hepatitis C virus genotype 4 infected adults with compensated cirrhosis (AGATE-I Part 2)**

# Complete Eligibility Criteria

*Inclusion Criteria*

1. Male or female at least 18 years of age at time of Screening.
2. Screening laboratory result indicating HCV genotype 4 infection.
3. Chronic HCV-infection prior to study enrolment. Chronic HCV-infection is defined as one of the following:

- Positive for anti-HCV Ab or HCV RNA >1000 IU/mL at least 6 months before Screening, and positive for HCV RNA and anti-HCV Ab at the time of Screening; or
- HCV RNA >1000 IU/mL at the time of Screening with a liver biopsy consistent with chronic HCV-infection (or a liver biopsy performed prior to enrolment with evidence of chronic hepatitis C disease).

1. Per local standard practice, documentation of cirrhosis by one of the following methods:

- Previous histologic diagnosis of cirrhosis on liver biopsy, e.g., Metavir Fibrosis Score of >3 (including 3 – 4 or 3/4), Ishak score of >4 or on a liver biopsy conducted during Screening; or
- A transient elastography (e.g., FibroScan^®^) score ≥14·6 kPa within 6 months of Screening or during the Screening Period; or
- A screening FibroTest >0·72 and APRI > 2.

Patients with a FibroScan result that is ≥12·5 kPa and <14·6 KPa; or a FibroTest result that is ≤0·72 and an APRI >2; or a FibroTest result that is >0·72 and an APRI ≤2 must have a liver biopsy performed prior to Screening showing evidence of cirrhosis, or in the absence of an available biopsy result prior to Screening, may undergo a liver biopsy during Screening to confirm cirrhosis. A patient with an exclusionary FibroTest but inclusionary FibroScan (or vice versa), is not eligible without a qualifying biopsy.

1. Compensated cirrhosis defined as a Child-Pugh Score of ≤6 at Screening.
2. For Arm C: Patient has never received antiviral treatment (including interferon/ribavirin) for hepatitis C infection (treatment-naïve patient), or patient must have documentation that they were adherent to prior IFN/RBV therapy and meet one of the following categories (treatment-experienced patient):

- Null responder:
  - received at least 10 weeks of IFN/RBV for the treatment of HCV and failed to achieve a 2 log10 IU/mL reduction in HCV RNA at Week 12 (patients will be considered to meet this definition if the lack of treatment response was documented between Weeks 10 – 16 of treatment); or
  - received at least 4 weeks of IFN/RBV for the treatment of HCV and achieved a <1 log_10_ IU/mL reduction in HCV RNA at Week 4 (patients will be considered to meet this definition if the lack of treatment response was documented after ≥25 days of treatment);
- Partial Responder: received at least 20 weeks of IFN/RBV for the treatment of HCV and achieved ≥2 log_10_ reduction in HCV RNA at Week 12 (Weeks 10 – 16), but failed to achieve HCV RNA undetectable at the end of treatment;
- Relapser: received at least 36 weeks of IFN/RBV for the treatment of HCV and was undetectable at or after the end of treatment, but HCV RNA was detectable within 52 weeks of treatment follow-up.

HCV RNA levels that serve as documentation to support the type of prior non-response should have been obtained in relation to the previous interferon/ribavirin treatment. Interferon/ribavirin therapy must have been completed no less than 2 months prior to the Screening Visit.

For Arm D: Patient must have prior treatment experience with SOF/pegIFN/RBV or SOF/RBV and meet one of the following categories:

- Prior SOF breakthrough/non-responder: HCV RNA detectable at the end of treatment with SOF/pegIFN/RBV or SOF/RBV;
- Prior SOF relapser: achieved HCV RNA undetectable at end of a prior treatment course SOF/pegIFN/RBV or SOF/RBV, but HCV RNA was detectable within 52 weeks following completion of therapy.

HCV RNA levels that serve as documentation to support the type of prior non-response should have been obtained in relation to the previous SOF/pegIFN/RBV or SOF/RBV treatment. SOF/pegIFN/RBV or SOF/RBV therapy must have been completed no less than 2 months prior to the Screening Visit.

1. Without evidence of hepatocellular carcinoma (HCC) as indicated by a negative ultrasound, computed tomography (CT) scan or magnetic resonance imaging (MRI) within 3 months prior to Screening or a negative ultrasound at Screening. Patients who have an ultrasound with results suspicious of HCC followed by a subsequent negative CT or MRI of the liver will be eligible for enrolment into the study.
2. If female, a patient who is:

- practicing total abstinence from sexual intercourse (minimum 1 complete menstrual cycle)
- sexually active with female partner(s) only
- not of childbearing potential, defined as:
  - postmenopausal for at least 2 years prior to Screening (defined as amenorrheic for longer than 2 years, age appropriate, and confirmed by a follicle-stimulating hormone [FSH] level indicating a postmenopausal state), or
  - surgically sterile (defined as bilateral tubal ligation, bilateral oophorectomy or hysterectomy) or has a vasectomized partner(s);
- of childbearing potential and sexually active with male partner(s):
  - currently using at least one effective method of birth control at the time of Screening, and
  - agree to practice two effective methods of birth control while receiving study drugs (as outlined in the patient information and consent form or other patient information documents), starting with Study Day 1 and for 7 months after stopping study drug, or as directed by the local ribavirin label.

(Note: Estrogen-containing hormonal contraceptives, including oral, injectable, implantable, patch and ring varieties, may not be used during study drug treatment).

1. Males who are not surgically sterile who are sexually active with female partner(s) of childbearing potential must agree to practice two effective forms of birth control (as outlined in the consent form or other patient information documents) throughout the course of the study, starting with Study Day 1 and for 7 months after stopping study drug, or as directed by the local ribavirin label.
2. Patients must be able to understand and adhere to the study visit schedule and all other protocol requirement and must voluntarily sign and date an informed consent form, approved by an Institutional Review Board (IRB)/Independent Ethics Committee (IEC), prior to the initiation of any screening or study specific procedures (see page 15 of supplement for list of IECs).

*Exclusion Criteria*

1. History of severe, life-threatening or other significant sensitivity to any drug.
2. Females who are pregnant or breastfeeding, or planning to become pregnant within 7 months (or per local RBV label) after their last dose of study drug, or males whose partners are pregnant or planning to become pregnant within 7 months (or per local RBV label) after their last dose of study drug.
3. Positive test result at Screening for Hepatitis B surface antigen (HbsAg) or anti-human immunodeficiency virus antibody (HIV Ab) confirmed by Multispot HIV-1/HIV-2 Rapid Test.
4. Use of any medications listed in the **Table** below, as well as any other medications that are contraindicated for use with either ritonavir or RBV, within 2 weeks of study drug administration including but not limited to:

| **Medications Contraindicated for Use with the Study Regimen** | | |
| --- | --- | --- |
| - Alfuzosin - Astemizole - Carbamazepine - Dihydroergotamine - Efavirnez - Ergotamine - Ergonovine | - Estrogen-Containing - Medications for Systemic Use - Fusidic Acid - Lovastatin - Methylergonovine - Midazolam (oral) - Phenobarbital - Phenytoin | - Pimozide - Rifampin - **Sildenafil**** - Simvastatin - St. John's Wort - Terfenadine - Triazolam |
| ** When used for the treatment of pulmonary arterial hypertension.  Note: Not all medications contraindicated with ritonavir and ribavirin are listed above. Refer to the most current package inserts or product labelling of ritonavir and ribavirin for a complete list of contraindicated medications. | | |

1. Use of known strong inducers of cytochrome P450 3A (CYP3A) (e.g., phenobarbital, rifampin, carbamazepine, St. John's Wort) within 2 weeks prior to initial dose of study drug.
2. Clinically significant abnormalities or co-morbidities (i.e., high risk of variceal bleeding), other than HCV infection and/or any reason in the opinion of the investigator that will make the patient an unsuitable candidate for this study or treatment with ribavirin.
3. Any current or past clinical evidence of Child-Pugh B or C classification or clinical history of liver decompensation, including ascites (noted on physical exam), variceal bleeding, or hepatic encephalopathy.
4. Current enrolment in another interventional clinical study, previous enrolment in this study, or previous use of any protease inhibitor, non-nucleoside polymerase inhibitor, or NS5a inhibitor, either investigational or commercially available (including previous exposure to ABT-450 or ombitasvir), or receipt of any investigational product within 6 weeks prior to study drug administration.
5. History of solid organ transplant.
6. HCV genotype performed during screening indicating unable to genotype or co-infection with any other HCV genotype.
7. Prior therapy with direct-acting antiviral agents for the treatment of HCV, including but not limited to, telaprevir, boceprevir, daclatasvir, simeprevir, and sofosbuvir.
8. Screening laboratory analyses showing any of the following abnormal laboratory results:

- Alanine aminotransferase (ALT) >7 × upper limit of normal (ULN)
- Aspartate aminotransferase (AST) >7 × ULN
- Albumin <2·8 g/dL
- International normalized ratio (INR) >2·3. Patients with a known inherited blood disorder and INR >2·3 may be enrolled with permission of the AbbVie Study Designated Physician.
- Calculated creatinine clearance (using Cockcroft-Gault method) <30 mL/min
- Haemoglobin ≤ lower limit of normal (LLN)
- Platelets <50,000 cells per mm^3^
- Indirect bilirubin >1·5 mg/dL
- Total bilirubin ≥3·0 mg/dL

1. Serum Alpha Fetoprotein (sAFP) > 100 ng/mL at Screening.
2. Confirmed presence of hepatocellular carcinoma (HCC) indicated on imaging techniques such as computed tomography (CT) scan or magnetic resonance imaging (MRI) within 3 months prior to Screening or on an ultrasound performed at Screening (a positive ultrasound result will be confirmed with CT scan or MRI).
3. Any cause of liver disease other than chronic HCV-infection, including but not limited to the following:

- Hemochromatosis
- Alpha-1 antitrypsin deficiency
- Wilson's disease
- Autoimmune hepatitis
- Alcoholic liver disease
- Drug-related liver disease

Steatosis and steatohepatitis on a liver biopsy coincident with HCV-related changes would not be considered exclusionary unless the steatohepatitis is considered to be the primary cause of the liver disease.

# Definition of Prior Treatment Experience

IFN/RBV treatment-experienced patients must have had documentation that they were adherent to prior interferon/ribavirin therapy and meet one of the following categories:

1. Null responder:
   1. Received at least 10 weeks of IFN/RBV for the treatment of HCV and failed to achieve a 2 log10 IU/mL reduction in HCV RNA at Week 12 (patients will be considered to meet this definition if the lack of treatment response was documented between Weeks 10 – 16 of treatment); or
   2. Received at least 4 weeks of IFN/RBV for the treatment of HCV and achieved a < 1 log10 IU/mL reduction in HCV RNA at Week 4 (patients will be considered to meet this definition if the lack of treatment response was documented after ≥ 25 days of treatment).
2. Partial Responder: received at least 20 weeks of IFN/RBV for the treatment of HCV and achieved ≥ 2 log10 reduction in HCV RNA at Week 12 (Weeks 10 – 16), but failed to achieve HCV RNA undetectable at the end of treatment.
3. Relapser: received at least 36 weeks of IFN/RBV for the treatment of HCV and was undetectable at or after the end of treatment, but HCV RNA was detectable within 52 weeks of treatment follow-up.

SOF/pegIFN/RBV or SOF/RBV treatment-experienced patients must have had documentation that they were adherent to prior therapy and meet one of the following categories:

1. Prior SOF breakthrough/non-responder: HCV RNA detectable at the end of treatment with SOF/pegIFN/RBV or SOF/RBV
2. Prior SOF relapser: achieved HCV RNA undetectable at end of a prior treatment course SOF/pegIFN/RBV or SOF/RBV, but HCV RNA was detectable within 52 weeks following completion of therapy.

# AGATE-I Protocol Guidelines for Ribavirin Dose Modifications

Reductions in haemoglobin are a well characterized effect of ribavirin exposure (1). All patients received weight-based ribavirin at the start of treatment, with dose modifications allowed during the study per guidelines below. For patients with reductions in haemoglobin levels, hematologic growth factors (e.g., erythropoietin) or blood transfusions were permitted at the discretion of the investigator.

| **Haemoglobin Value** | **Ribavirin Dosing Recommendation** |
| --- | --- |
| **Patients without history of cardiac disease and creatinine clearance ≥ 50 mL/min** | |
| < 10 g/dL and ≥ 8·5 g/dL | Reduce ribavirin dose (follow ribavirin label for dose reductions). Study drugs may be continued. If haemoglobin increases to ≥ 10 g/dL, then the ribavirin dose may then be increased, with gradual dose increases in 200 mg increments towards original dose. |
| < 8·5 g/dL | Interrupt ribavirin treatment and manage the patient as medically appropriate. If haemoglobin increases to ≥ 8·5 g/dL then ribavirin dosing may resume. |
| **Patients with history of stable cardiac disease and creatinine clearance ≥ 50 mL/min** | |
| Haemoglobin decrease of ≥ 2 g/dL  during any 4-week treatment period | Reduce ribavirin dose (follow ribavirin label for dose reductions). If a subsequent haemoglobin result is greater than the level that triggered the dose reduction, then the ribavirin dose may then be increased, with gradual dose increases in 200 mg increments. |
| < 12 g/dL after a 4-week ribavirin dose reduction | Interrupt ribavirin treatment and manage the patient as medically appropriate. If haemoglobin increases to ≥ 12 g/dL then ribavirin dosing may resume. |

For patients with renal impairment, initial ribavirin dosing is based on the following creatinine clearance values:

| **Creatinine clearance value** | **Ribavirin Dose** |
| --- | --- |
| 30 – 50 mL/min | Alternating doses, 200 mg and 400 mg every other day |
| < 30 mL/min | 200 mg daily |
| Haemodialysis | 200 mg daily |

For patients with creatinine clearance < 50 mL/min, if haemoglobin levels were < 10 g/dL then ribavirin dosing was interrupted, and the patient was managed as medically appropriate. If haemoglobin levels increased to ≥ 10 g/dL, then ribavirin dosing could be resumed. If creatinine clearance levels increased to ≥ 50 mL/min, then investigators deferred to the guidance above.

**RNA extraction and quantification**

Plasma was processed within 6 hours of collection and stored frozen until required for testingat a central laboratory. RNA extraction and the subsequent quantitation of RNA levels in plasma samples was carried out using the COBAS AmpliPrep/COBAS TaqMan^®^ HCV Test, v2.0 (HC2), P/N 05480442190 per the manufacturer’s instructions on the COBAS AmpliPrep and COBAS TaqMan^®^ Instruments respectively. COBAS TaqMan^®^ real-time reverse transcriptase-PCR assay v.2.0 (Roche Molecular Diagnostics, Pleasanton, CA, USA) has a lower limit of detection (LLOD) of 12 IU/mL in genotype 4 and a lower limit of quantification (LLOQ) of 25 IU/Ml (2).

**HCV genotyping and phylogenetic analysis**

Plasma samples for HCV genotype and subtype were collected at screening. Genotype was assessed using the Versant^®^ HCV Genotype Inno-LiPA Assay, version 2.0 (LiPA; Siemens Healthcare Diagnostics, Tarrytown, NY, USA). The Versant HCV genotype Inno-LiPA assay was performed according to the manufacturer’s instructions (Siemens, Part No. 06714844). Phylogenetic analysis was conducted by amplifying and sequencing a 329-nucleotide region of NS5B from available patient baseline samples, followed by neighbor-joining phylogenetic analysis to determine genotype 4 subtype (3,4). Neighbor-joining phylogenetic analysis was conducted with the HKY85 nucleotide substitution model and 1000 bootstrapping replicates, using Geneious 9 software (Biomatters Ltd., Auckland, New Zealand).

**Outcomes**

On-treatment virologic failure was defined as a confirmed increase from nadir in HCV RNA (defined as two consecutive HCV RNA measurements > 1 log_10_ IU/mL above nadir) at any time point during treatment; confirmed HCV RNA at LLOQ or higher at any point after HCV RNA less than LLOQ during treatment; or failure to achieve HCV RNA less than LLOQ in patients who received at least 6 weeks of treatment. Post-treatment relapse was defined as confirmed HCV RNA greater than or equal to LLOQ between end of treatment and 12 weeks after last dose of study drug among patients completing treatment and with HCV RNA less than LLOQ at the end of treatment. Patients with on-treatment virologic failure were to discontinue study treatment.

Additional outcomes included the SVR rates in Arm D of patients who failed to achieve SVR with either prior sofosbuvir and pegylated interferon plus ribavirin, or sofosbuvir plus ribavirin treatment, to evaluate efficacy after 24 weeks’ treatment in this patient group.

The sample size for the study was determined based on the power for the primary endpoint comparison. With a total sample size of about 60 patients per treatment arm (Arms A, B and C) and assuming that 90% of the patients in each arm will achieve SVR12, this study has greater than 95% power to demonstrate superiority with a two-sided 97.5% lower confidence bound greater than 67% (based on the normal approximation of a single binomial proportion in a one-sample test for superiority using EAST 6.2). All patients were monitored for Child-Pugh score changes and for all adverse events that occurred from the first day of study drug administration until 30 days’ post treatment. Events were coded using the Medical Dictionary for Regulatory Activities (MedRA) Version 18.1, graded for severity, and assessed for causality as related to the study drugs by the investigator.

**Statistical Analysis**

The study was designed to test the hypothesis that percentage of treatment-naive and interferon-experienced HCV genotype 4-infected patients with compensated cirrhosis treated with ombitasvir/paritaprevir/ritonavir co-administered with ribavirin for 24 weeks achieving SVR12 is superior to a clinically relevant threshold (based on historical SVR rates for HCV genotype 4-infected patients treated with pegylated interferon plus ribavirin.

# Threshold Calculation

Different studies have reported SVR rates for patients infected with HCV genotype 4 and treated with pegylated interferon and ribavirin in Middle East and worldwide populations. The majority of patients were treatment-naive. Limited data are available in HCV genotype 4-infected patients with cirrhosis. A summary of the results of those studies is presented in the **Table**.

| **SVR Rates in Treatment-Naive HCV Genotype 4-Infected Patients Treated with Pegylated Interferon and Ribavirin** | | | |
| --- | --- | --- | --- |
|  | **Sustained virologic response** | |  |
| **Reference** | **n/N** | **%** | **Population** |
| Kamal SM, et al^5^ | 66/96 | 69% | Egyptian patients only |
| Hasan F, et al^6^ | 45/66 | 66% | Middle Eastern patients only |
| Derbala MF, et al^7^ | 25/38 | 66% | Middle Eastern patients only |
| Kamal SM, et al^8^ | 30/50 | 60% | Egyptian patients only |
| Thakeb F, et al^9^ | 35/51 | 69% | Egyptian patients only |
| Fried MW, et al^10^ | 10/13 | 77% | Worldwide patients |

The weighted average of the SVR rates for these studies is approximately 67%. The rate of 67% should be conservative, because the 67% was based on a mix of HCV genotype 4-infected patients with or without cirrhosis and the expectation is that the SVR rate for patients with cirrhosis would be lower than the SVR rate for the population without cirrhosis

The percentage of patients with SVR12 within Arm C was calculated with a two-sided 97.5% confidence interval (CI) using the Wilson score method, and the lower boundary was compared with the defined threshold. The lower bound of the 97.5% CI of the SVR12 rate had to be greater than 67% in order for the regimen to be considered superior to pegylated interferon plus ribavirin. The overall two-sided significance level of 0.05 was split between Part I (Arms A and B) and Part II (Arm C) using a Bonferroni corrected alpha level of 0.025 for each part.

In addition, SVR12 and the percentages of patients with on-treatment virologic failure during treatment and post-treatment relapse through Post-Treatment Week 12 in Arm D and two-sided 95% CI using the Wilson score method were calculated.

A sensitivity analysis in which SVR12 data were assessed excluding patients who were categorized as either having prematurely discontinued study drug with no on-treatment virologic failure or missing follow-up data in the SVR12 window was carried out for Arm C. The number and proportion of patients achieving SVR12 were reported with 95% Wilson score CIs.

The proportion difference of patients achieving SVR12 (with 95% confidence intervals) between Arms B and A, and between Arms B and C were calculated using stratum-adjusted Mantel–Haenszel proportion and continuity-corrected variance, adjusting for interferon/ribavirin treatment history (treatment naive or treatment experienced). In addition, percentages (with two-sided 95% CI using Wilson score method) of patients with on-treatment virologic failure during treatment and post-treatment relapse were calculated and summarized.

The mean change from baseline was summarized for FibroTest score, and mean change from baseline to Post-Treatment Week 12 in FibroTest scores were compared between Arms C and B from Part II using an analysis of covariance (ANCOVA) model with treatment arm as a factor and baseline score as a covariate.

Safety analyses were performed on the 24-week treatment groups on the safety population that includes all patients who receive at least one dose of study drug.

SAS software version 9.3 for the UNIX operating system was used for all analyses.

**Figure S1. Comparison of efficacy of ombitasvir, paritaprevir, and ritonavir, plus ribavirin, in patients with hepatitis C virus genotype 4 infection and compensated cirrhosis in Parts I and II.** Data are percentage for the SVR12 ITT analysis; bars represent 95% confidence intervals for each arm as calculated by the Wilson Score method. Proportional differences between groups were calculated using stratum-adjusted Mantel–Haenszel proportion and continuity-corrected variance, adjusting for interferon/ribavirin treatment history. ITT = intention-to-treat population; SVR12 = sustained virologic response at Post-Treatment Week 12. * Sensitivity analysis excluded patients who were categorized as either having “prematurely discontinued study drug with no on-treatment virologic failure” or “missing follow-up data in the SVR12 window.”


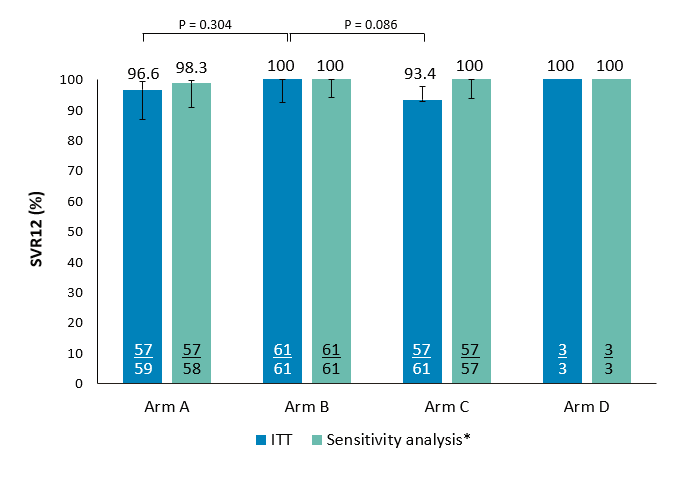


# Serious Adverse Events Reported in the AGATE-I, Part II Study

Serious adverse events reported by patients who received ombitasvir/paritaprevir/ritonavir with ribavirin for 24 weeks:

- One 78-year-old female patient reported a serious event of grade 4 acute liver toxicity, which was considered to be related to study drug. The patient discontinued study drug.
- One 59-year-old male patient experienced a serious event of myocardial infarction, which was considered not related to study drugs
- One 62-year-old male reported a serious event of suicidal thoughts, which was considered not related to study drugs

**Table: Adverse events and post-baseline laboratory abnormalities (safety population)**

|  | **Arm A**  **(n = 60*)** | **Arms B**  **(n = 60)** | **Arm C**  **(n = 61)** | **Arm D**  **(n = 3)** |
| --- | --- | --- | --- | --- |
| **Adverse events, n (%)** | | | | |
| **Any adverse events** | 48 (80%) | 56 (93%) | 54 (89%) | 3 (100%) |
| **Adverse events leading to study drug discontinuation** | 0 | 0 | 2 (3%) | 0 |
| **Severe adverse event** | 2 (3%) | 4 (7%) | 3 (5%) | 0 |
| **Serious adverse event** | 4 (7%) | 4 (7%) | 3 (5%) | 0 |
| **Death** | 0 | 1 (2%) | 0 | 0 |
| **Adverse events occurring in >10% of patients^†^** | | | | |
| **Fatigue** | 10 (17%) | 20 (33%) | 16 (26%) | 0 |
| **Asthenia** | 11 (18%) | 19 (32%) | 15 (25%) | 1 (33%) |
| **Headache** | 14 (23%) | 14 (23%) | 13 (21%) | 0 |
| **Anaemia** | 9 (15%) | 12 (20%) | 7 (12%) | 0 |
| **Pruritus** | 5 (8%) | 6 (10%) | 12 (20%) | 1 (33%) |
| **Nausea** | 6 (10%) | 8 (13%) | 5 (8%) | 1 (33%) |
| **Haemoglobin decreased** | 3 (5%) | 8 (13%) | 4 (7%) | 0 |
| **Dizziness** | 4 (7%) | 9 (15%) | 0 | 0 |
| **Insomnia** | 5 (8%) | 6 (10%) | 5 (8%) | 1 (33%) |
| **Myalgia** | 3 (5%) | 6 (10%) | 3 (5%) | 0 |
| **Post-baseline laboratory abnormalities, n/N_OBS (%)** | | | | |
| **Hemoglobin ≥ grade 2 (<10g/dL)** | 6/59 (10%) | 12/60 (20%) | 7/61 (12%) | 0/3 |
| **Hemoglobin ≥ grade 3 (<8g/dL)** | 0/59 | 1/60 (2%) | 0/61 | 0/3 |
| **Alanine aminotransferase**  **≥ grade 3 (>5 × ULN)** | 2/59 (3%) | 0/60 | 3/61 (5%) | 0/3 |
| **Aspartate aminotransferase**  **≥ grade 3 (>5 × ULN)** | 0/59 | 0/60 | 1/61 (2%) | 0/3 |
| **Total bilirubin ≥ grade 3 (≥3 × ULN)** | 5/59 (9%) | 3/60 (5%) | 5/61 (8%) | 0/3 |

n/N_OBS, indicates the number of patients with post-baseline value through the final treatment value for the respective parameter; ULN, upper limit of normal.
* One patient was originally randomly allocated to receive treatment for 16 weeks but only received 12 weeks of treatment and is included in the 12-week group (Arm A) for the safety analysis;

^†^ Adverse events occurring in >10% of patients in Arms A–C.

**References**

1. Banerjee D and Reddy KR. Review article: safety and tolerability of direct-acting antiviral agents in the new era of hepatitis C therapy. *Aliment Pharmacol Ther* 2016; 43(6):674–696.
2. Pas S, Molenkamp R, Schinkel J, et al. Performance evaluation of the new Roche cobas AmpliPrep/cobas TaqMan HCV test, version 2.0, for detection and quantification of hepatitis C virus RNA. *J Clin Microbiol* 2013; 51(1):238-242.
3. Koletzki D, Dumont S, Vermeiren H, Fevery B, De Smet P, Stuyver LJ. Development and evaluation of an automated hepatitis C virus NS5B sequence-based subtyping assay. *Clin Chem Lab Med* 2010; 48(8):1095-102.
4. Murphy D, Willems B, Deschênes M, Hilzenrat N, Mousseau R, and Sabbah S. Use of Sequence Analysis of the NS5B Region for Routine Genotyping of Hepatitis C Virus with Reference to C/E1 and 5′ Untranslated Region Sequences. *J Clin Microbiol* 2007; 45(4):1102–1112.
5. Kamal SM, El Tawil AA, Nakano T, et al. Peginterferon {alpha}-2b and ribavirin therapy in chronic hepatitis C genotype 4: impact of treatment duration and viral kinetics on sustained virological response. *Gut* 2005; 54(6): 858–66.
6. Hasan F, Asker H, Al-Khaldi J, et al. Peginterferon alfa-2b plus ribavirin for the treatment of chronic hepatitis C genotype 4. *Am J Gastroenterol* 2004; 99(9):1733–1737.
7. Derbala MF, Al Kaabi SR, El Dweik NZ, et al. Treatment of hepatitis C virus genotype 4 with peginterferon alfa-2a: impact of bilharziasis and fibrosis stage. *World J Gastroenterol* 2006; 12(35):5692–5698.
8. Kamal SM, El Kamary SS, Shardell MD, et al. Pegylated interferon alpha-2b plus ribavirin in patients with genotype 4 chronic hepatitis C: The role of rapid and early virologic response. *Hepatology* 2007; 46(6):1732–1740.
9. Thakeb F, Omar M, Bilharz T, El Awady M, Isshak S. Randomized controlled trial of peginterferon alfa-2a plus ribavirin for chronic hepatitis C virus-genotype 4 among Egyptian patients. *Hepatology* 2003; 38(4):278A.
10. Fried MW, Shiffman ML, Reddy KR, et al. Peginterferon alfa-2a plus ribavirin for chronic hepatitis C virus infection. *N Engl J Med* 2002; 347(13):975–982.

**International Ethics Committees (IECs) for study M11-665**

| **Name and Address of IEC/IRB** |
| --- |
| **Local:**  Azienda Universita di Padova  Comitato Etico per la Sperimentazione  c/o Nucleo Ricerca Clinica dell'A.O. di Padova  Via Giustiniani, 1  PADUA, 35128  Italy |
| **Local:**  The Johns Hopkins University Hospital  Office of Human Subjects Research – IRB  Reed Hall Suite B-130  1620 McElderry Street  BALTIMORE, MD 21205-1911  United States |
| **Central:**  CPP lle de France 8, Hopital Ambroise Pare  Laboratoire d'Anatomopathologie  9 avenue Charles de Gaulle  BOULOGNE BILLANCOURT, 92100  France |
| **Local:**  Landesamt fur Gesundheit und Soziales (LAGeSo)  Ethik-Kommission des Landes Berlin  Fehrbelliner Platz 1  BERLIN, 10707  Germany  **Central:**  Ethik-Kommission des Fachbereichs Medizin  der Johann Wolfgang Goethe-Universitat  Haus 1  Theodor-Stern-Kai 7  FRANKFURT, 60590  Germany |

| **Central:**  Hospital Universitarrio Puerta de Hierro Majadahonda  Secretaria Tecnica del CEIC (Entrada por Laboratorios-Banco de Sangre)  Planta 1a - Pasillo, Unidades Administrativas (peines F-G)  C/Joaquin Rodrigo, 2 (Entrada por Manuel de Falla, 1)  MAJADAHONDA (MADRID), 28222  Spain  **Local:**  Hospital Universitario La Fe  Secretaria del CEIC  Torre A, planta 7a  Bulevar Sur, s/n  VALENCIA, 46026  Spain |
| --- |
| **Local:**  IRCCS Inst Clinico Humanitas  Comitato Etico dell`IRCCS Inst Clinico Humanita  Via A. Manzoni, 56  ROZZANO (MILAN),  Italy |
| **Central:**  Hospital Universitarrio Puerta de Hierro Majadahonda  Secretaria Tecnica del CEIC (Entrada por Laboratorios-Banco de Sangre)  Planta 1a - Pasillo, Unidades Administrativas (peines F-G)  C/Joaquin Rodrigo, 2 (Entrada por Manuel de Falla, 1)  MAJADAHONDA (MADRID), 28222  Spain  **Local:**  CEIC Hospital Universitario Puerta de Hierro  Secretaria Tecnica del CEIC (Entrada por Laboratorios-Banco de Sangre)  Planta 1a - Pasillo, Unidades Administrativas (peines F-G)  C/Joaquin Rodrigo, 2 (Entrada por Manuel de Falla, 1)  MAJADAHONDA, MADRID, 28222  Spain |
| **Local:**  A.O.U. Citta della Salute e della Scienza di Turin  Comitato Etico Interaziendale  C. so Bramante, 88/90  TURIN, 10126  Italy |
| **Central:**  CPP lle de France 8, Hopital Ambroise Pare  Laboratoire d'Anatomopathologie  9 avenue Charles de Gaulle  BOULOGNE BILLANCOURT, 92100  France |
| **Central:**  National Ethics Committee (E.E.D)  National Organization of Medicines (E.O.F.)  284 Mesogeion Avenue,  HOLARGOS, 15562  Greece  **Local:**  General and Oncology Hospital of Kifisia Agioi Anargyroi  Hospital Scientific Committee  General and Oncology  Kaliftaki, Nea Kifisia  ATHENS, 14564  Greece |
| **Central:**  Institutional Review Board Services  Suite 300  372 Hollandview Trail  AURORA, ON L4G 0A5  Canada |
| **Local:**  University Health Network - Toronto Western Hospital  Research Ethics Board  10th Floor, Room 10-56  700 University Avenue  TORONTO, ON M5G 1Z5  Canada |
| **Central:**  Ethikkommission der Stadt Wien  MA 15, Schnirchgasse 12/2, Stiege 2, 1. Stock  VIENNA, 1030  Austria  **Local:**  Medical University of Vienna  Ethikkommission der Medizinischen Universitaet Wien  und des Allgemeinen Krankenhauses der Stadt Wien  Borschkegasse 8b/E06  VIENNA, 1090  Austria |
| **Local:**  Azienda Ospedaliero Universitaria di Parma  Comitato Etico per Parma  Via Gramsci 14  PARMA, 43100  Italy |
| **Central:**  Ethik-Kommission des Fachbereichs Medizin  der Johann Wolfgang Goethe-Universitat  Haus 1  Theodor-Stern-Kai 7  FRANKFURT, 60590  Germany  **Local:**  Ethik-Kommission der Medizinischen Fakultaet  Robert-Koch-Str. 9-11  ESSEN, 45147  Germany |
| **Central:**  Ethikkommission der Stadt Wien  MA 15, Schnirchgasse 12/2, Stiege 2, 1. Stock  VIENNA, 1030  Austria |
| **Central:**  Quorum Review IRB  Suite 800  1501 Fourth Avenue  SEATTLE, WA 98101  United States |
| **Central:**  CPP lle de France 8, Hopital Ambroise Pare  Laboratoire d'Anatomopathologie  Hospital Ambroise Pare  9 Avenue Charles de Gaulle  BOULOGNE BILLANCOURT, 92100  France |
| **Central:**  Commission d'Ethique Biomedicale  UCL Saint Luc  Avenue Hippocrate, 55.14 Tour Harvey, Niveau 0  BRUSSELS, 1200  Belgium |
| **Central:**  National Ethics Committee (E.E.D)  National Organization of Medicines (E.O.F.)  284 Mesogeion Avenue,  HOLARGOS, 15562  Greece  **Local:**  General Hospital of Rhodes  Hospital Scientific Committee  Agioi Apostoloi Street Rhodes, Dodekanisos  RHODES, 85100  Greece |
| **Central:**  Ethikkommission der Stadt Wien  MA 15, Schnirchgasse 12/2, Stiege 2, 1. Stock  VIENNA, 1030  Austria |
| **Central:**  CPP lle de France 8, Hopital Ambroise Pare  Laboratoire d'Anatomopathologie  9 avenue Charles de Gaulle  BOULOGNE BILLANCOURT, 92100  France |
| **Local:**  IRCCS Inst Clinico Humanitas  Comitato Etico dell`IRCCS Inst Clinico Humanita  Via A. Manzoni, 56  ROZZANO (MILAN),  Italy |
| **Central:**  Commission d'Ethique Biomedicale  UCL Saint Luc  Avenue Hippocrate, 55.14 Tour Harvey, Niveau 0  BRUSSELS, 1200  Belgium |
| **Central:**  Commission d'Ethique Biomedicale  UCL Saint Luc  Avenue Hippocrate, 55.14 Tour Harvey, Niveau 0  BRUSSELS, 1200  Belgium |
| **Local:**  Kaiser Permanente  Southern California Institutional Review Board  4th Floor  393 East Walnut Street  PASADENA, CA 91188  United States |
| **Local:**  Hospital Universitario La Paz  CEIC - Area 5, Planta octava del hospital general  despacho 818-819  Paseo de la Castellana 261  MADRID, 28046  Spain  **Central:**  Hospital Universitarrio Puerta de Hierro Majadahonda  Secretaria Tecnica del CEIC (Entrada por Laboratorios-Banco de Sangre)  Planta 1a - Pasillo, Unidades Administrativas (peines F-G)  C/Joaquin Rodrigo, 2 (Entrada por Manuel de Falla, 1)  MAJADAHONDA (MADRID), 28222  Spain |
| **Local:**  Azienda Ospedaliero Universitaria di Parma  Comitato Etico per Parma  Via Gramsci 14  PARMA, 43126  Italy |
| **Central:**  National Ethics Committee (E.E.D)  National Organization of Medicines (E.O.F.)  284 Mesogeion Avenue,  HOLARGOS, 15562  Greece  **Local:**  General Hospital of Athens "Laiko"  Hospital Scientific Committee  17 Agiou Thoma Street  ATHENS, 11527  Greece |
| **Central:**  CPP lle de France 8, Hopital Ambroise Pare  Laboratoire d'Anatomopathologie  9 avenue Charles de Gaulle  BOULOGNE BILLANCOURT, 92100  France |
| **Local:**  A.O.U. Citta della Salute e della Scienza di Turin  Comitato Etico Interaziendale  A.O.Ordine Mauriziano- A.S.L TO1  C. so Bramante, 88/90  TURIN, 10126  Italy |
| **Local:**  Hospital Parc de Salut del Mar  CEIC Institut Municipal d'Investigacio Medica (IMIM)  Secretaria Tecnica CEIC-IMAS 1 Planta  C/Dr. Alguader, 88 - Edifici PRBB  BARCELONA, 08003  Spain  **Central:**  Hospital Universitarrio Puerta de Hierro Majadahonda  Secretaria Tecnica del CEIC (Entrada por Laboratorios-Banco de Sangre)  Planta 1a - Pasillo, Unidades Administrativas (peines F-G)  C/ Joaquin Rodrigo, 2 (Entrada por Manuel de Falla, 1)  MAJADAHONDA (MADRID), 28222  Spain |
| **Central:**  Hospital Universitarrio Puerta de Hierro Majadahonda  Secretaria Tecnica del CEIC (Entrada por Laboratorios-Banco de Sangre)  Planta 1a - Pasillo, Unidades Administrativas (peines F-G)  C/Joaquin Rodrigo, 2 (Entrada por Manuel de Falla, 1)  MAJADAHONDA (MADRID), 28222  Spain  **Local:**  Comite Etico de Investigacion Clinica de Galicia  Division de Farmacia y Productos Sanitarios  Edificio Administrative San Lazaro, s/n  La Coruna  SANTIAGO DE COMPOSTELA, 15703  Spain |
| **Local:**  UBC Clinical Research Ethics Board  Room 210, Research Pavillion  828 West 10th Avenue  VANCOUVER, BC V5Z 1L8  Canada |
| **Central:**  Ethik-Kommission des Fachbereichs Medizin  der Johann Wolfgang Goethe-Universitat  Haus 1  Theodor-Stern-Kai 7  FRANKFURT, 60590  Germany |
